# A Social Ecological Approach to Hazardous Alcohol Use among Flemish Higher Education Students

**DOI:** 10.3390/ijerph17218288

**Published:** 2020-11-09

**Authors:** Robert Tholen, Edwin Wouters, Koen Ponnet, Sara De Bruyn, Guido Van Hal

**Affiliations:** 1 Centre for Population, Family and Health, University of Antwerp, 2000 Antwerp, Belgium; edwin.wouters@uantwerpen.be (E.W.); sara.debruyn@uantwerpen.be (S.D.B.); 2 Department of Communication Studies, imec-mict, Ghent University, 9000 Ghent, Belgium; koen.ponnet@ugent.be; 3 Department of Epidemiology and Social Medicine, Social Epidemiology and Health Policy, University of Antwerp, 2610 Wilrijk, Belgium; guido.vanhal@uantwerpen.be

**Keywords:** alcohol consumption, higher education students, student health, environmental factors, quantitative research

## Abstract

Hazardous use of alcohol is a global public health concern. Statistics suggest that this is particularly common in Europe, and among higher education students. Although it has been established that various factors—ranging from the individual to the overarching societal level—are associated with misuse of alcohol, few studies take multiple levels of influence into account simultaneously. The current study, therefore, used a social ecological framework to explore associations between variables from multiple levels of influence and the hazardous use of alcohol. Data were obtained from a representative sample of higher education students from Flanders, Belgium (*n* = 21,854), and explored using hierarchical multiple regression analyses. The results demonstrated that the individual, interpersonal, organizational, community, and policy levels, were all associated with risky alcohol consumption. When devising interventions, policymakers should, therefore, take into consideration that variables from multiple levels of influence are at play. Students’ capacities to change or maintain their alcohol consumption behaviors may be undermined if social settings, overarching environments, social norms, and policies are not conducive to their motivations and social expectations.

## 1. Introduction

High-volume consumption of alcohol and risky single occasion drinking (RSOD) are common practices among higher education students [1,2]. Past research demonstrates that these practices have been around for a number of decades [3], and are more prominent among those attending higher education than in their age-matched peers [4,5]. Research also suggests that consumption of alcohol—excessive and binge drinking, in particular [6,7,8]—has become normalized among young people attending higher education, and inextricably linked to student life [6,9]. Such alcohol practices, however, are potentially dangerous as they can produce a large number of adverse effects, ranging from individual short- and long-term health consequences (e.g., injuries, diseases) [10], to adverse (secondhand) outcomes in social situations (e.g., drunk driving, sexual assault) [11]. Importantly, studies consistently show that high-volume alcohol consumption in late adolescence is likely to continue into adulthood and also increases the likelihood of alcohol problems occurring (including dependence) [12].

Although most research has focused on the US, scientific attention aimed at students’ alcohol use in Europe has increased [8,9]. Alcohol use appears to be widespread in Europe, as the EU countries, plus Switzerland and Norway, continue to have the highest global level of alcohol consumption per capita [13]. In addition, since there are major differences in social, legal, and cultural contexts (e.g., school systems, campus living, legal drinking age, integration of alcohol consumption in daily life) between Europe and the US, it is of vital importance to continue Europe-focused research endeavors [8,9]. 

The current study focuses on Flanders, one of three regions in Belgium, by using a representative sample of Flemish students. In Belgium, alcohol is considered to be part of the culture and is associated with numerous social activities like dinners or gatherings with friends and family [14]. The cultural significance of alcohol in Belgium is further demonstrated by UNESCO’s inscription of Belgian beer culture on the Representative List of the Intangible Cultural Heritage of Humanity in 2016. In Belgium as a whole, people consume more alcohol per capita (11.1 L) [15] than the European average (9.8 L) [16], and the prevalence of RSOD (defined by the WHO as consumption of at least 60 g of pure alcohol on at least one occasion in the past 30 days) is also above average—particularly among people in the age range 15–24 years [17]. Belgium also has one of the highest proportions of risky users among adolescents in Europe [18], and rates of binge drinking among higher education students did not decline over a period of six years [19]. Furthermore, one study estimated that close to half (45%) of the social cost of substance misuse in Belgium could be attributed to alcohol [20]. Notably, alcohol statistics suggest that hazardous use of alcohol (high-volume alcohol consumption, as well as RSOD) is more common among Flemish young adults in the conventional student age range of 17 to 24 in comparison to Belgian young adults in the same age range, as well as the general population (aged 15 and over) in both Belgium and Flanders [21]. Considering the ubiquity of hazardous alcohol use among higher education students and its associated risks, this topic remains an important public health concern.

### A Social Ecological Approach

One of the key social ecological models was described by Bronfenbrenner [22]. He argued that to understand human behavior, one has to take into consideration the immediate environment, as well as the broader formal and informal social contexts within which people are embedded. If this is not done, one cannot fully grasp the complexity of the behavior that is being studied, as human behavior both affects and is affected by the social environment which surrounds us [22]. This ecological perspective was further developed into a framework aimed at guiding behavioral health interventions by McLeroy and others [23]. Five hierarchical levels of influence are identified, which are considered to impact health-related behaviors. The (1) individual level refers to characteristics of (or related to) the individual, such as sociodemographic information (e.g., sex and age). The (2) interpersonal level includes characteristics related to (in)formal social networks and support systems, such as family (e.g., education level of parents) and peers (e.g., closeness to friends). The (3) organizational level takes into account the influences of social institutions on health-related behaviors (e.g., membership of social organizations). The (4) community level includes characteristics of, or is related to, the environments in which people spend time—for students, the higher education environment is one of these, if not the primary environment—and their influences on health-related behaviors (e.g., social norms about alcohol behaviors). Finally, the (5) policy level includes policies, laws, and public health awareness campaigns.

Numerous studies have addressed potential factors that contribute to hazardous drinking among higher education students. A common observation across these studies is that various levels of influence exist, ranging from the individual level to the overarching societal level [2,4], in line with the premises of the social ecological model. One of the shortcomings of most studies, however, is that they focus on only one or two levels of influence. Usually, this includes individual-level characteristics, as well as characteristics of the higher education environment (e.g., social involvement, social norms, campus policy) [1,24,25,26]. 

There is, thus, a dearth of research taking into account multiple levels of influence when approaching the complex social problem of hazardous alcohol use among higher education students. Such studies would allow researchers to compare the relative influence of these levels and enable them to estimate the effect of predictors, while taking into account an encompassing array of variables. Considering that research addressing multiple levels of influence may simultaneously lead to the most effective interventions [2,4,9,27,28,29], such studies would be well suited to provide more empirically-based knowledge as input for the further development of multi-level interventions. We found two studies incorporating three or more levels of influence. One American study distinguished between individual-level, interpersonal-level and community-level characteristics [30], and one Thai study applied the social ecological framework [31]. While the social ecological approach is well suited for this goal, to our knowledge, no similar research has been performed in the European context. Therefore, the current article examines the hazardous use of alcohol in a representative sample of Flemish higher education students, using a social ecological framework. We answer the following research question: To what extent are the individual, interpersonal, organizational, community and policy levels of influence associated with hazardous alcohol use among Flemish higher education students?

## 2. Materials and Methods 

The analyses are based on a substance use data collection project entitled ‘Head in the clouds?’ among students from all higher education institutions in Flanders (Belgium) [32]. An online cross-sectional survey was conducted between the beginning of March and the end of April 2017. Higher education institutions were able to use their preferred channels (e.g., the digital learning platform, mailings) to inform students about the survey. Prior to participation, students were informed about the goal and the contents of the survey and were assured that the data would be collected anonymously. If they agreed to participate, they were asked to provide their written informed consent. Following their participation, the students received contact information from the Flemish drug helpline (De DrugLijn) in case they had personal questions concerning the topics that were covered. The study was approved by the Ethics Committee of the Medicine and Health Sciences Faculty of Ghent University on 3 February 2017 (EC UZG 2017/0113).

Institutions with a response rate of less than 5% were excluded from the dataset (this corresponds to 820 respondents). These students were removed from the anonymized dataset that we were given access to. The dataset used for analyses included 35,221 higher education students (15.9% response rate). For the current study, students aged 17 to 24 were selected in order to reflect the conventional student age range (*n* = 31,847). Next, we selected students (1) who reported having consumed alcohol in the year prior to questioning and (2) who responded to all items included in the dependent variable (*n* = 25,103). Only those students who had no missing values on all variables were included in the statistical models, resulting in a final sample of 21,854 students between the ages of 17 and 24 years.

Representativity of the data was assessed by comparing the sample distribution to the population distribution by means of information made available by the Flemish Ministry of Education and Training [33]. The χ^2^ tests in Table 1 indicate that women (χ^2^ = 622.06) and first year students (χ^2^ = 81.47) were overrepresented in our sample. These differences were adjusted using post-stratification weights. The weighted mean age of the students in our final sample is 20.62 (SD = 1.76).

### 2.1. Measures

Our dependent variable is the short form of the Alcohol Use Disorders Identification Test, AUDIT-Consumption (AUDIT-C), which measures the quantity and frequency of drinking. The AUDIT-C was originally developed as a screening instrument for practitioners to identify individuals at risk of developing alcohol problems and to consequently refer them for further alcohol assessments or interventions [34]. Cutoff scores are applied to distinguish risky from non-risky drinkers dichotomously. Given that the goal of the current study is to broaden our understanding of the multiple levels of influence that contribute to the hazardous use of alcohol, rather than to identify at-risk individuals, the AUDIT-C is used as a continuous measure of drinking severity with higher scores indicating more hazardous drinking [35]. The scale has been shown to have sound psychometric qualities when used with student populations [36,37]. 

The AUDIT-C consists of three questions in which students are asked to think back over the past 12 months and indicate (1) how many times they drank alcohol, (2) how many glasses of alcohol they usually drank per day, and (3) how many times they drank six or more glasses of alcohol on one single occasion. A glass of alcohol was defined as a standard glass for each type of alcoholic beverage (beer (<6% alcohol), strong beer (≥6% alcohol), wine, fortified drink, spirit) and the corresponding quantity in centiliters (25, 33, 10, 5 and 3.5 cl.). The scale had good reliability in our sample (*α* = 0.82). We used the sum scores to create a single item (mean = 4.53; SD = 2.69; range = 0–12).

#### 2.1.1. Individual Level

The following variables at the individual level were included: sex (men = 0; women = 1), study year (first year = 0; higher year = 1), living situation (at home = 0; away from home (during the week) = 1), employment (no job = 0; job = 1), and age of onset alcohol use. Study year was included as it was the only information available with which to account for the time exposed to the higher education environment [1]. In Belgium, it is common for students with student residences to live with their parents during the weekends. Since they live in their residences during the week, we considered these students to be living away from home. Age of onset alcohol use was centered around 0 (mean = 14.79; SD = 1.86). Furthermore, the importance of religion (“How important is religion in your life?”) was rated on a 5-point Likert scale. Finally, mental distress was measured using the anxiety and depression subscale from the General Health Questionnaire containing four items (GHQ-12) [38]. This scale had an acceptable level of reliability (α = 0.76). Age was not included, as our preliminary analyses demonstrated a non-linear relationship between age and AUDIT-C.

#### 2.1.2. Interpersonal level

On the interpersonal level, we included parental educational attainment (0 = no higher education degree among parents; 1 = higher education degree among parents). Next, students were asked to indicate whether they would talk with (1) family members, and/or (2) friends about alcohol problems (“Imagine you or your friend has a problem with alcohol or other drugs. With whom would you talk about this?”) (0 = no; 1 = yes). Students were also asked to rate the trustworthiness of people (“Do you think that, in general, most people can be trusted (10) or that you can’t be careful enough in dealing with people (0)”).

#### 2.1.3. Organizational Level

On the organizational level, students were asked whether they had an affiliation with the following organizations: (1) (board) membership student association; (2) membership sports club/team; (3) membership or group leader of youth movement (0 = no; 1 = yes). A group leader of a youth movement supervises social activities for a group of children during the weekends.

#### 2.1.4. Community Level

The following questions concerning social norms were put to all students: “During the academic year (excluding exam periods), how often in the past 12 months do you think (1) an average male student drank six or more alcoholic consumptions in 2 h; (2) an average student drank sufficient alcohol to feel drunk?” (0 = never; 1 = once a month; 2 = 2–3 times a month; 3 = once a week; 4 = twice a week; 5 = 3–4 times a week; 6 = 5–6 times a week; 7 = daily). The last question surrounding social norms that was included is as follows: “In the past 12 months, how many alcoholic consumptions do you think an average student drank on an average day during the academic year (excluding exam periods)” (0 = none; 1 = 1 glass; 2 = 2 glasses; 3 = 3–4 glasses; 4 = 5–6 glasses; 5 = 7–8 glasses; 6 = 9–11 glasses; 7 = 12–15 glasses; 8 = 16–18 glasses; 9 = 19–24 glasses; 10 = 25 glasses or more). Similar to the AUDIT-C questions, a glass of alcohol was defined as a standard glass for each type of alcoholic beverage. Finally, an item on the alcohol theme in the study curriculum was added (“Is the alcohol and drug theme addressed in the study curriculum?”) (0 = no; 1 = yes).

#### 2.1.5. Policy Level

Students were asked whether they had participated in a public health awareness campaign on alcohol use, the annually organized Tournée Minérale. In this campaign, people are challenged to give up alcohol for one month in February. Similar temporary alcohol abstinence challenges are organized elsewhere (e.g., Dry January in the UK). For clarity purposes, we henceforth refer to this campaign as Dry February. The answer categories were: (1) Participation in Dry February (regardless of success, since there were very few students who participated and failed) (reference category); (2) unfamiliar with Dry February; (3) intentionally did not participate in Dry February.

### 2.2. Analytic Strategy

To address our research questions, we first performed descriptive and bivariate analyses. The mean differences in AUDIT-C scores for all independent variables were tested by means of independent samples t-tests or ANOVA tests. For continuous variables, Spearman rank correlations were calculated. Our next step was to run hierarchical linear regression analyses. Prior to running these analyses, we checked all of the required assumptions to allow the use of the ordinary least squares (OLS) method. This procedure is outlined in Supplementary 1. In order to establish the appropriate strategy for handling missing values, we undertook a number of steps. Our own analysis of missing data patterns combined with Little’s test (χ^2^ = 138.01, df = 97, *p* = 0.004) demonstrated that our data are MAR. We imputed ten datasets and ran pooled regression analyses. These results (N = 25,103) were nearly identical to the regression analyses including respondents with no missing values (N = 21,854), with the exception of very few, minor differences. The latter analyses were used in the current study. The results of the pooled regression analyses are available upon request.

Five models were tested. The first model includes only the individual level. Each level was added successively until we incorporated all levels in the fifth and final model. All analyses were performed with IBM SPSS Statistics 24 (IBM Corp., Armonk, NY, USA).

## 3. Results

The results of our bivariate analyses are presented in Table 2. Since all bivariate analyses provided significant results, we will only discuss the most relevant outcomes. On the individual level, we found that men had significantly higher scores on the AUDIT-C (t(18,847.230) = 52.97, *p* < 0.001), as did students not living at home (t(20,950.358) = −26.59, *p* < 0.001). Furthermore, students with an earlier age of onset of alcohol use showed more hazardous drinking (ρ = −0.28, *p* < 0.001). On the interpersonal level, the results showed that students willing to talk to family members about alcohol problems had significantly lower scores (t(21,335.427) = 19.93, *p* < 0.001), while students willing to talk to friends about these problems had significantly higher scores (t(6273.227) = −19.13, *p* < 0.001). The results on the organizational level demonstrate that students with (board) memberships of student associations (t(14,215.859) = −22.26, *p* < 0.001), and members or group leaders of youth movements (t(21,852) = −29.69, *p* < 0.001), had significantly higher AUDIT-C scores. On the community level, we found that students who thought that other students exhibited more hazardous alcohol consumption were more likely to show hazardous alcohol behavior themselves. In particular, the held social norm surrounding the amount of alcohol consumption was associated with higher AUDIT-C scores (ρ = 0.32, *p* < 0.001). Finally, on the policy level, we found that students who did not participate in Dry February (DF) had significantly higher AUDIT-C scores (F(2, 21,851) = 1389.907, *p* < 0.001).

The results of the final regression analysis are presented in Table 3. All variables on the individual level, except study year (*β* = −0.01, *p* = 0.13), explained a significant amount of the variance in the dependent variable, AUDIT-C. Male gender (*β* = −0.34, *p* < 0.001), living away from home (*β* = 0.19, *p* < 0.001) and having an early onset age of alcohol use (*β* = −0.18, *p* < 0.001) were the main explanatory variables on the individual level. Together, the variables accounted for 20.6% of the variance (F(7,21,845) = 810.15, *p* < 0.001). 

The variables on the interpersonal level were significantly associated with AUDIT-C. The willingness to talk to family members about an alcohol or drug-related problem was associated with less hazardous alcohol use (*β* = −0.10, *p* < 0.001), while a willingness to talk to friends was associated with more hazardous alcohol use (*β* = 0.09, *p* < 0.001). The interpersonal level accounted for an additional 2.4% explained variance (F(4, 21,841) = 173.94, *p* < 0.001).

Being a member or group leader of a youth movement (*β* = 0.18, *p* < 0.001) and being a (board) member of a student association (*β* = 0.10, *p* < 0.001) were the primary explanatory variables of hazardous alcohol use on the organizational level. The additional explained variance of this level of influence was 4.0% (F(3, 21,838) = 396.91, *p* < 0.001).

Not all variables on the community level were significantly associated with hazardous alcohol use. Whether or not students thought that alcohol or other drugs were addressed in their study curriculum was not significantly associated with scores on the AUDIT-C (*β* = 0.01, *p* = 0.31). Students’ social norms were significantly associated with hazardous alcohol use, in particular, norms relating to the binge drinking behavior of men (*β* = 0.12, *p* < 0.001) and norms relating to the consumption amount (*β* = 0.20 *p* < 0.001). The community level accounted for an additional 8.5% of the explained variance (F(4, 21,834) = 718.74, *p* < 0.001).

The policy variable was significantly associated with hazardous alcohol use. Students who participated were less likely to engage in hazardous alcohol consumption in comparison to those who intentionally did not participate in Dry February (*β* = 0.26, *p* < 0.001). Adding the policy level explained an additional variance of 4.4% (F(2, 21,832) = 812.30, *p* < 0.001).

## 4. Discussion

The current study used a social ecological framework to explore how multiple levels of influence affect hazardous alcohol use among Flemish higher education students [22,23]. We found that the individual, interpersonal, organizational, community, as well as policy levels, were all associated with risky alcohol consumption. The final model explained 39.9% of the variance in AUDIT-C scores. Taking into consideration all levels of influence, the primary explanatory variables for hazardous alcohol use were male sex (*β* = −0.28, *p* < 0.001) (individual level), not participating in Dry February (*β* = 0.26, *p* < 0.001) (policy level), the held social norms surrounding alcohol consumption (*β* = 0.20, *p* < 0.001) (community level), being a member or group leader of a youth movement (*β* = 0.14, *p* < 0.001) (organizational level), and the age of onset of alcohol use (*β* = −0.13, *p* < 0.001) (individual level).

On the *individual* level, our main results correspond with prior academic work showing that hazardous use of alcohol use is more likely to occur among male students [2,9,27,39] and students with a lower age of onset of alcohol use [2,9,40,41]. In considering the role of gender, it is important to note that the definition of binge drinking used in the AUDIT-C (six or more drinks on one occasion) differs slightly from the Flemish definition (four or more drinks for women and six or more drinks for men within a time frame of 2 h) [42]. 

The variables included on the *interpersonal* level did not play a large relative role in explaining risky alcohol behavior. The largest relative association was found for students who said they would talk to family members about alcohol problems, which is in line with earlier research demonstrating good parent-child communication as a protective factor [4,27,43]. Parental educational attainment did not contribute very much to our model, in contrast to earlier studies [8,9,27,44]. One explanation could be that the majority of students had at least one parent with a higher education degree (78%), which may have led to an underestimation of the true association.

On the *organizational* level, it was found that being a member or a group leader of a youth movement was associated with the hazardous use of alcohol. This association is difficult to fully understand, since students were not asked to define the type of youth movement they were affiliated with. Furthermore, we found very little research involving this association. One twin study indicated that involvement in social activities may protect against the development of hazardous alcohol use [45]. However, participants were only asked about involvement in social activities during (young) adolescence (ages 12 to 17) and not young adulthood, and also about broader social involvement, beyond youth movements. More studies are needed to fully understand the mechanisms driving this association.

Next, on the *community* level, we found that students’ social norms surrounding consumption amount had a relatively strong impact. This result corresponds with earlier research, showing that students with perceptions of more permissive norms surrounding alcohol consumption were more likely to engage in RSOD and high-volume alcohol consumption [2,8]. This association is alarming, considering that students have a tendency to overestimate their peers’ alcohol consumption [2,9,46]. On the other hand, students that do make accurate estimations about the alcohol use of their peers tend to display less hazardous use of alcohol themselves [47,48]. 

Finally, on the *policy* level, we found that participation in a public health awareness campaign was associated with hazardous alcohol use. This finding is similar to earlier research performed in the UK [49,50]. Considering our data are cross-sectional, it could be that a self-selection effect is at play, in that those students who already exhibit less hazardous alcohol behavior were more likely to voluntarily abstain from alcohol for a month. 

The presented findings are subject to some limitations. Since the data are cross-sectional in nature, causal inferences cannot be made. Furthermore, as all data are self-reported measures, the common-method variance may affect the results. Due to the anonymity requirements of the use of the data, we had no further information on where students were from and which higher education institution they were attending. Had this information been available, multi-level analyses could have been performed, with the addition of relevant objective measures (e.g., alcohol outlet density and frequency [51]) to enrich the current analysis. In addition, information concerning specific policies enforced by particular higher education institutions could have been added. Since higher-level laws and policies regarding alcohol-related themes were the same for all students in the sample, we were not able to fully capture the policy level of influence. It is also important to keep in mind that the data may suffer from self-selection bias; students exhibiting high-risk alcohol consumption may have dropped out of their institution. Nevertheless, this study is one of, if not the first, to demonstrate the applicability of the social ecological framework in addressing the hazardous use of alcohol in a representative sample of higher education students. Our results provide researchers and policymakers with knowledge that may assist the further development of multi-level interventions.

Over the years, a number of interventions have been designed, implemented, and tested among specific higher education populations. Among the most effective interventions are brief motivational interventions, in particular, personalized feedback and normative reeducation [52,53,54,55]. These interventions are located at the individual level of influence, and as such, do not directly impact—if at all—upon other levels of influence. Despite their proven effectiveness, merely aiming to change students’ perceptions and motivations may prove inadequate if social settings, environments, and policies hinder students from maintaining their healthy behaviors [28]. Environment-focused interventions, on the other hand, have been found to be less effective if not combined with interventions aimed at the individual level [52]. Hazardous alcohol use may, thus, be best targeted by means of multi-level interventions [2,4,9,27,28,29]. Based on the results of the current study, we suggest that the above-mentioned characteristics, in particular, should be taken into consideration when devising comprehensive policy initiatives to reduce hazardous alcohol use. 

Ideally, explorative studies with the aim of informing multi-level interventions should acquire representative student data that make it possible to include objectively measured characteristics at the organizational and community level. Unfortunately, such data are hard to come by; nevertheless, studies, such as the current one, enable universities, as well as policymakers, to make more informed decisions. There is certainly a need for more studies and intervention designs that are critically informed, and can address multiple levels of influence simultaneously. Much is still to be learned about which ecological mixes [52] are best suited to the acquisition of optimally informed, and structurally sound, interventions targeting the complex social problem of hazardous alcohol use among higher education students.

## 5. Conclusions

The hazardous use of alcohol among higher education students in Flanders is a public health concern. Using a social ecological framework, the current study demonstrates that multiple levels of influence are at play in predicting hazardous drinking. While evidence-based interventions are available, these are mostly aimed at addressing only a single level of influence. Students’ capacity to maintain responsible levels of drinking may be undermined if the social settings in which they are embedded, and the overarching environments, social norms, and policies, are not conducive to their motivations and social expectations. The results presented here may guide policymakers toward the development of effective multi-level interventions.

## Figures and Tables

**Table 1:** Chi-square difference tests between the Flemish higher education population in the academic year, 2016–2017 (born between 1992 and 1999), and survey data of students aged 17 to 24 years, stratified by sex and study year [33].

Stratification Criteria		Total Population (*n* = 214,826) % (*n*)	Sample (*n* = 21,854) % (*n*)	χ^2^ Test
*Study year by sex*				χ^2^ = 705.45 df = 3 *p* < 0.001
Men	First year	9.7% (20,913)	8.8% (1930)	
	Higher year	35.7% (76,618)	28.2% (6156)	
Women	First year	11.7% (25,133)	15.1% (3294)	
	Higher year	42.9% (92,162)	47.9% (10,474)	
*Sex*	Men	45.4% (97,531)	37.0% (8086)	χ^2^ = 622.06 df = 1 *p* < 0.001
	Women	54.6% (117,295)	63.0% (13,768)	
*Study year*	First year	21.4% (46,046)	23.9% (5224)	χ^2^ = 81.47 df = 1 *p* < 0.001
	Higher year	78.6% (168,780)	76.1% (16.630)	

**Table 2:** Descriptive statistics with differences in AUDIT-C scores (*n* = 21,854).

*Level of Influence* Variables	Range	Mean (SD)	Statistic	Standardized Effect Size	Sample (Unweighted)
*Individual level*					
Sex	Men (0)	5.54 (2.82)	t(18,847.230) = 52.97 ***	*g* = −0.73	8086
	Women (1)	3.69 (2.26)			13,768
Study year	First year (0)	4.43 (2.67)	t(21,852) = −2.74 *p* = 0.006	*g* = 0.05	5224
	Higher year (1)	4.55 (2.70)			16,630
Living situation	At home (0)	3.99 (2.58)	t(20,950.358) = −26.59 ***	*g* = 0.36	9549
	Not at home (1)	4.95 (2.70)			12,305
Job	No job (0)	4.38 (2.69)	t(21,852) = −11.07 ***	*g* = 0.16	14,128
	Job (1)	4.80 (2.67)			7726
Importance religion	0–4		ρ = −0.13 ***		
Mental distress	0–4		ρ = −0.08 ***		
Age onset alcohol use (mean centered)	−10–8		ρ = −0.28 ***		
*Interpersonal level*					
Parent(s) higher education degree	No (0)	4.16 (2.67)	t(21,852) = –10.83 ***	*g* = 0.18	4884
	Yes (1)	4.63 (2.69)			16,970
Talk to family about alcohol problem	No (0)	4.91 (2.74)	t(21,335.427) = 19.93 ***	*g* = –0.27	10,229
	Yes (1)	4.19 (2.60)			11,625
Talk to friends about alcohol problem	No (0)	3.82 (2.61)	t(6273.227) = −19.13 ***	*g* = 0.33	4141
	Yes (1)	4.69 (2.68)			17,713
Trustworthiness of people	0–10		ρ = 0.10 ***		
*Organizational level*					
Member/board student association	No (0)	4.24 (2.62)	t(14,215.859) = −22.26 ***	*g* = 0.32	14,607
	Yes (1)	5.10 (2.74)			7247
Member sport club or team	No (0)	4.46 (2.66)	t(12,503.985) = −5.94 ***	*g* = 0.09	15,270
	Yes (1)	4.69 (2.75)			6584
Member/group leader youth movement	No (0)	4.25 (2.63)	t(21,852) = −29.69 ***	*g* = 0.49	17,199
	Yes (1)	5.55 (2.66)			4655
*Community level*					
Alcohol/drug theme in the curriculum?	No (0)	4.55 (2.71)	t(7512.693) = 3.21 *p* = 0.001	*g* = −0.05	17,075
	Yes (1)	4.41 (2.63)			4779
Social norm binge drinking men	0–7		ρ = 0.22 ***		
Social norm drunkenness	0–7		ρ = 0.21 ***		
Social norm consumption amount	0–10		ρ = 0.32 ***		
*Policy level*					
Participation in Dry February (DF)	Unfamiliar	3.47 (2.40)	F(2, 21,851) = 1389.91 ***	*f* = 0.36	2487
	Didn’t participate	5.03 (2.61)			16,302
	Participated	2.59 (2.18)			3065

* *p* < 0.05; ** *p* < 0.01; *** *p* < 0.001; t = independent samples t or Welch t when homogeneity of variances cannot be assumed; ρ = Spearman rank correlation; F = one-way ANOVA F; *g* = Hedges’ *g*; *f* = Cohen’s *f.*

**Table 3:** Final multiple regression model predicting AUDIT-C (*n* = 21,854).

	B	SE	ß	95% CI for B
Constant	0.47 ***	0.09		0.30–0.65
*Individual level*				
Sex	−1.51 ***	0.03	−0.28	−1.57–−1.45
Study year	0.00	0.04	0.00	−0.07–0.07
Living situation	0.64 ***	0.03	0.12	0.58–0.70
Job	0.49 ***	0.03	0.09	0.43–0.55
Importance religion	−0.19 ***	0.02	−0.07	−0.22–−0.17
Mental distress	−0.03 **	0.01	−0.02	−0.05–−0.01
Age onset alcohol use (mean centered)	−0.19 ***	0.01	−0.13	−0.21–−0.18
*Interpersonal level*				
Parent(s) HE degree	0.09 **	0.04	0.01	0.02–0.16
Talk to family about alcohol problem	−0.42 ***	0.03	−0.08	−0.47–−0.36
Talk to friends about alcohol problem	0.44 ***	0.04	0.06	0.37–0.51
Trustworthiness of people	0.06 ***	0.01	0.04	0.04–0.07
*Organizational level*				
Member/board student association	0.50 ***	0.03	0.09	0.44–0.56
Member sport club or team	0.14 ***	0.03	0.02	0.08–0.20
Member/group leader youth movement	0.89 ***	0.04	0.14	0.82–0.96
*Community level*				
Alcohol/drug theme in the curriculum?	0.04	0.03	0.01	−0.03–0.11
Social norm binge drinking men	0.26 ***	0.01	0.12	0.23–0.29
Social norm drunkenness	0.06 ***	0.02	0.03	0.03–0.09
Social norm consumption amount	0.38 ***	0.01	0.20	0.36–0.40
*Policy level*				
Participated in DF ^^^ (ref.)				
Unfamiliar with DF	0.62 ***	0.06	0.07	0.51–0.73
Didn’t participate in DF	1.60 ***	0.04	0.26	1.51–1.68
Adjusted R square	39.9%			

* *p* < 0.05; ** *p* < 0.01; *** *p* < 0.001; *B* = unstandardized coefficients; SE = standard error for *B*; *ß* = standardized coefficients; CI = confidence interval for *B*; ^^^ DF = Dry February.

## Supplementary Materials

The following is available online at https://www.mdpi.com/1660-4601/17/21/8288/s1. Supplementary 1: Validity of the use of the ordinary least squares (OLS) method.

## References

[1] Lorant V., Nicaise P., Soto V.E., d’Hoore W. (2013). Alcohol drinking among college students: College responsibility for personal troubles. BMC Public Health.

[2] Ham L.S., Hope D.A. (2003). College students and problematic drinking: A review of the literature. Clin. Psychol. Rev..

[3] Straus R., Bacon S.D. (1953). Drinking in College.

[4] Merrill J.E., Carey K.B. (2016). Drinking over the lifespan: Focus on college ages. Alcohol Res. Curr. Rev..

[5] Carter A.C., Brandon K.O., Goldman M.S. (2010). The college and noncollege experience: A review of the factors that influence drinking behavior in young adulthood. J. Stud. Alcohol Drugs.

[6] Supski S., Lindsay J., Tanner C. (2016). University students’ drinking as a social practice and the challenge for public health. Crit. Public Health.

[7] Saether S.M.M., Knapstad M., Askeland K.G., Skogen J.C. (2019). Alcohol consumption, life satisfaction and mental health among Norwegian college and university students. Addict. Behav. Rep..

[8] Kuntsche E., Rehm J., Gmel G. (2004). Characteristics of binge drinkers in Europe. Soc. Sci. Med..

[9] Wicki M., Kuntsche E., Gmel G. (2010). Drinking at European universities? A review of students’ alcohol use. Addict. Behav..

[10] World Health Organization (2019). Global Status Report on Alcohol and Health 2018.

[11] Wechsler H., Nelson T.F. (2008). What we have learned from the Harvard School of Public Health College Alcohol Study: Focusing attention on college student alcohol consumption and the environmental conditions that promote it. J. Stud. Alcohol Drugs.

[12] McCambridge J., McAlaney J., Rowe R. (2011). Adult consequences of late adolescent alcohol consumption: A systematic review of cohort studies. PLoS Med..

[13] World Health Organization (2019). Status Report on Alcohol Consumption, Harm and Policy Responses in 30 European Countries 2019.

[14] Vlaams Expertisecentrum Alcohol en Andere Drugs (VAD) Alcohol en Jongeren. https://www.vad.be/artikels/detail/alcohol-en-jongeren.

[15] World Health Organization Total (Recorded + Unrecorded) Alcohol per Capita Consumption by Country. https://apps.who.int/gho/data/node.main.A1029SDG3?lang=en.

[16] World Health Organization Regional Alcohol per Capita (15+) Consumption by WHO Region. https://apps.who.int/gho/data/node.main.A1035?lang=en.

[17] World Health Organization (2019). Belgium: Alcohol Consumption, Harm and Policy Responses Fact Sheet.

[18] Braker A.B., Soellner R. (2016). Alcohol drinking cultures of European adolescents. Eur. J. Public Health.

[19] Tavolacci M., De Bruyn S., Van der Heijde C., Vonk P., Ladner J., Van Hal G. (2019). Tobacco smoking and binge drinking among university students in three European countries, 2009–2017: Marie-Pierre Tavolacci. Eur. J. Public Health.

[20] Lievens D., Vander Laenen F., Verhaeghe N., Putman K., Pauwels L., Hardyns W., Annemans L. (2017). Economic consequences of legal and illegal drugs: The case of social costs in Belgium. Int. J. Drug Policy.

[21] Drieskens S., Charafeddine R., Demarest S., Gisle L., Tafforeau J., Van der Heyden J. Health Interview Survey, Belgium, 1997–2001–2004–2008–2013–2018: Health Interview Survey Interactive Analysis. https://hisia.wiv-isp.be/.

[22] Bronfenbrenner U. (1977). Toward an experimental ecology of human development. Am. Psychol..

[23] McLeroy K.R., Bibeau D., Steckler A., Glanz K. (1988). An ecological perspective on health promotion programs. Health Educ. Quart..

[24] Delgado-Lobete L., Montes-Montes R., Vila-Paz A., Cruz-Valiño J.-M., Gándara-Gafo B., Talavera-Valverde M.-Á., Santos-del-Riego S. (2020). Individual and Environmental Factors Associated with Tobacco Smoking, Alcohol Abuse and Illegal Drug Consumption in University Students: A Mediating Analysis. Int. J. Environ. Res. Public Health.

[25] Van Damme J., Hublet A., De Clercq B., McAlaney J., Van Hal G., Rosiers J., Maes L., Clays E. (2016). Context matters: Student-perceived binge drinking norms at faculty-level relate to binge drinking behavior in higher education. Addict. Behav..

[26] Oh S.S., Ju Y.J., Jang S.I., Park E.C. (2020). Self-reported campus alcohol policy and college alcohol consumption: A multilevel analysis of 4592 Korean students from 82 colleges. Subst. Abus. Treat. Prev. Policy.

[27] Kuntsche E., Kuntsche S., Thrul J., Gmel G. (2017). Binge drinking: Health impact, prevalence, correlates and interventions. Psychol. Health.

[28] Sallis J.F., Owen N., Fisher E. (2008). Ecological models of health behavior. Health Behavior: Theory, Research, and Practice.

[29] Sudhinaraset M., Wigglesworth C., Takeuchi D.T. (2016). Social and cultural contexts of alcohol use: Influences in a social–ecological framework. Alcohol Res. Curr. Rev..

[30] Haardörfer R., Windle M., Fairman R.T., Berg C.J. (2020). Longitudinal changes in alcohol use and binge-drinking among young-adult college students: Analyses of predictors across system levels. Addict. Behav..

[31] Vantamay S. (2009). Alcohol consumption among university students: Applying a social ecological approach for multi-level preventions. Southeast Asian J. Trop. Med. Public Health.

[32] Van Damme J., Thienpondt A., Rosiers J., De Bruyn S., Soyez V., Sisk M., Van Hal G., Deforche B. (2018). In Hogere Sferen?, Volume 4: Een Onderzoek Naar Het Middelengebruik bij Vlaamse Studenten.

[33] Vlaamse Ministerie van Onderwijs en Vorming Statistisch Jaarboek van het Vlaams Onderwijs 2016–2017. https://onderwijs.vlaanderen.be/nl/statistisch-jaarboek-van-het-vlaams-onderwijs-2016-2017-0.

[34] Bush K., Kivlahan D.R., McDonell M.B., Fihn S.D., Bradley K.A. (1998). The AUDIT alcohol consumption questions (AUDIT-C): An effective brief screening test for problem drinking. Arch. Intern. Med..

[35] Rubinsky A.D., Dawson D.A., Williams E.C., Kivlahan D.R., Bradley K.A. (2013). AUDIT-C scores as a scaled marker of mean daily drinking, alcohol use disorder severity, and probability of alcohol dependence in a U.S. general population sample of drinkers. Alcohol Clin. Exp. Res..

[36] Barry A.E., Chaney B.H., Stellefson M.L., Dodd V. (2013). Evaluating the psychometric properties of the AUDIT-C among college students. J. Subst. Use.

[37] Verhoog S., Dopmeijer J.M., de Jonge J.M., van der Heijde C.M., Vonk P., Bovens R., de Boer M.R., Hoekstra T., Kunst A.E., Wiers R.W. (2020). The Use of the Alcohol Use Disorders Identification Test—Consumption as an Indicator of Hazardous Alcohol Use among University Students. Eur. Addict. Res..

[38] Graetz B. (1991). Multidimensional properties of the general health questionnaire. Soc. Psych. Psych. Epid..

[39] Tavolacci M.P., Boerg E., Richard L., Meyrignac G., Dechelotte P., Ladner J. (2016). Prevalence of binge drinking and associated behaviours among 3286 college students in France. BMC Public Health.

[40] De Bruyn S., Wouters E., Ponnet K., Van Damme J., Maes L., Van Hal G., Task Force substance use in Flemish universities and colleges (2018). Problem drinking among Flemish students: Beverage type, early drinking onset and negative personal & social consequences. BMC Public Health.

[41] Caamano-Isorna F., Adkins A., Aliev F., Moure-Rodriguez L., Dick D.M. (2020). Population Attributable Fraction of Early Age of Onset of Alcohol Use in Alcohol Abuse and Dependence: A 3-Year Follow-Up Study in University Students. Int. J. Environ. Res. Public Health.

[42] Amrani L., De Backer L., Dom G. (2013). Adolescent binge drinking: Neurocognitive consequences and gender differences. Tijdschrift voor Psychiatrie.

[43] Stephenson M., Barr P., Ksinan A., Aliev F., Latvala A., Viken R., Rose R., Kaprio J., Dick D., Salvatore J.E. (2020). Which adolescent factors predict alcohol misuse in young adulthood? A co-twin comparisons study. Addiction.

[44] Humensky J.L. (2010). Are adolescents with high socioeconomic status more likely to engage in alcohol and illicit drug use in early adulthood?. Subst. Abus. Treat. Prev. Policy.

[45] Wichers M., Gillespie N.A., Kendler K.S. (2013). Genetic and Environmental Predictors of Latent Trajectories of Alcohol Use from Adolescence to Adulthood: A Male Twin Study. Alcohol. Clin. Exp. Res..

[46] Perkins H.W. (2002). Social norms and the prevention of alcohol misuse in collegiate contexts. J. Stud. Alcohol Suppl..

[47] Stock C., McAlaney J., Pischke C., Vriesacker B., Van Hal G., Akvardar Y., Orosova O., Kalina O., Guillen-Grima F., Bewick B.M. (2014). Student estimations of peer alcohol consumption: Links between the Social Norms Approach and the Health Promoting University concept. Scand. J. Public Health.

[48] McAlaney J., Helmer S.M., Stock C., Vriesacker B., Van Hal G., Dempsey R.C., Akvardar Y., Salonna F., Kalina O., Guillen-Grima F. (2015). Personal and perceived peer use of and attitudes toward alcohol among university and college students in seven EU countries: Project SNIPE. J. Stud. Alcohol Drugs.

[49] de Visser R.O., Robinson E., Bond R. (2016). Voluntary temporary abstinence from alcohol during “Dry January” and subsequent alcohol use. Health Psychol..

[50] de Visser R.O., Robinson E., Smith T., Cass G., Walmsley M. (2017). The growth of ‘Dry January’: Promoting participation and the benefits of participation. Eur. J. Public Health.

[51] Gruenewald P.J., Remer L.G., LaScala E.A. (2014). Testing a social ecological model of alcohol use: The California 50-city study. Addiction.

[52] Stellefson M., Barry A.E., Stewart M., Paige S.R., Apperson A., Garris E., Russell A. (2019). Resources to Reduce Underage Drinking Risks and Associated Harms: Social Ecological Perspectives. Health Promot. Pract..

[53] Neale Z.E., Salvatore J.E., Cooke M.E., Savage J.E., Aliev F., Donovan K.K., Hancock L.C., Dick D.M. (2018). The Utility of a Brief Web-Based Prevention Intervention as a Universal Approach for Risky Alcohol Use in College Students: Evidence of Moderation by Family History. Front. Psychol..

[54] Su J., Hancock L., Wattenmaker McGann A., Alshagra M., Ericson R., Niazi Z., Dick D.M., Adkins A. (2018). Evaluating the effect of a campus-wide social norms marketing intervention on alcohol-use perceptions, consumption, and blackouts. J. Am. Coll. Health.

[55] Plotnikoff R.C., Costigan S.A., Kennedy S.G., Robards S.L., Germov J., Wild C. (2019). Efficacy of interventions targeting alcohol, drug and smoking behaviors in university and college students: A review of randomized controlled trials. J. Am. Coll. Health.

