# Supplementary material for: A Social Ecological Approach to Hazardous Alcohol Use among Flemish Higher Education Students
Source: Int J Environ Res Public Health. 2020 Nov 9;17(21):8288. doi: 10.3390/ijerph17218288 (PMC7720127; doi:10.3390/ijerph17218288)
Supplement: Supplementary file 1 [file ijerph-17-08288-s001.zip › supplementary 1.docx]

Supplementary 1: Validity of the use of the ordinary least squares (OLS) method

To ensure an OLS method could validly be used, a number of assumptions were tested:

*Linear relationship between the dependent and independent variables:* In the regression model, there was a linear relationship between all independent variables combined and the dependent variable. When checking all variables, we found a non-linear relationship between age and AUDIT-C total score (F(6, 21846) = 2.539, *p* = 0.019). Therefore, age was not included in our models.

*Independence of residuals:* Since the Durbin-Watson value was very close to 2 (*d* = 1.962), this assumption was met.

*Homoscedasticity:* We found that our data displayed homoscedasticity (see Figure A1).


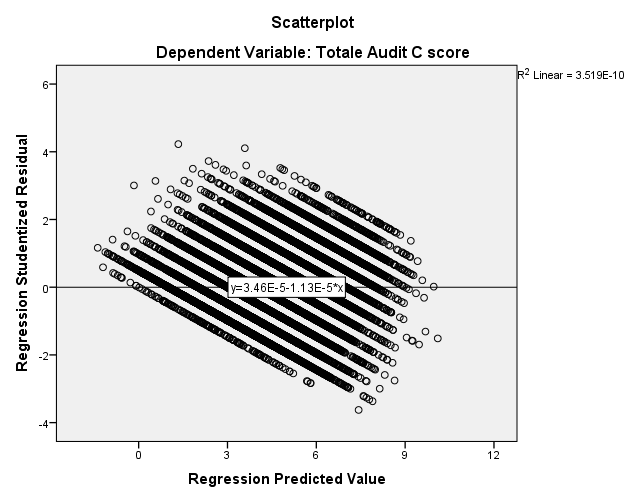


**Figure A1.** Scatterplot of dependent variable

*Multicollinearity:* The correlation table showed no correlations higher than 0.70 or lower than -0.70. The VIF values ranged from 1.01 to 1.88, so no indications of multicollinearity were found.

*Outliers, high leverage points and influential cases:* We looked for outliers using scores on Cook’s distance, standardized residuals, centered leverage values, DFBETA scores and the scatterplot featured above. Outliers were individually checked. We found no strange coding mishaps or contradictory answers, only large differences between actual and predicted scores on the dependent variables. All outliers appeared to be extreme observations from the tails of the normal distribution – and not influential – and were therefore retained in the model.

*The residuals of the regression should follow a normal distribution:* Both the histogram and the P-P Plot demonstrated that the residuals followed a normal distribution (see images below).


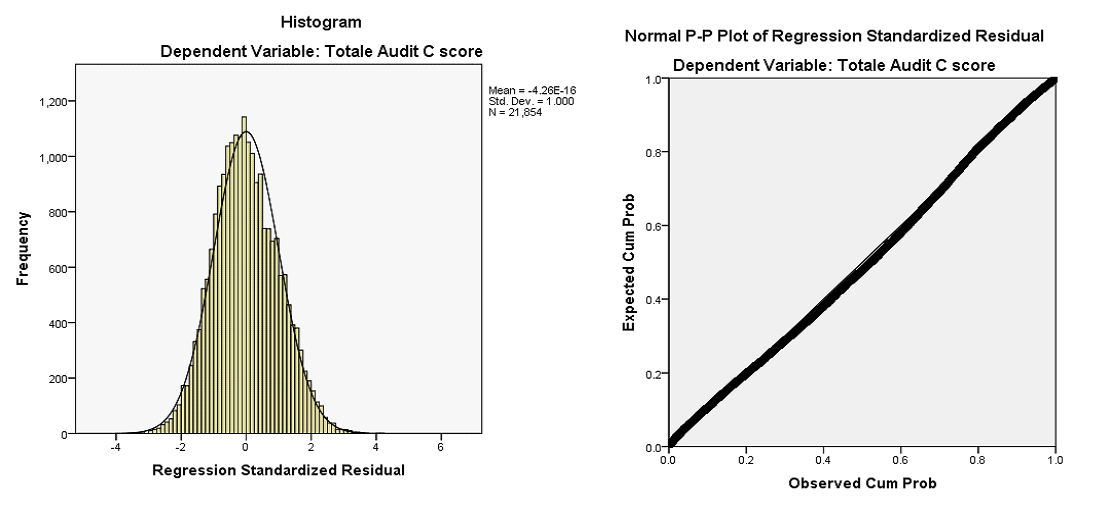


**Figure A2.** Histogram and P-Plot of the dependent variable

| 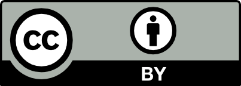 | © 2020 by the authors. Submitted for possible open access publication under the terms and conditions of the Creative Commons Attribution (CC BY) license (http://creativecommons.org/licenses/by/4.0/). |
| --- | --- |
